# Chest Radiography and Xpert MTB/RIF® Testing in Persons with Presumptive Pulmonary TB: Gaps and Challenges from a District in Karnataka, India

**DOI:** 10.1155/2020/5632810

**Published:** 2020-01-04

**Authors:** Manjula Kanakaraju, Sharath Burugina Nagaraja, Srinath Satyanarayana, Yella Ramesh Babu, Akshaya Kibballi Madhukeshwar, Somashekar Narasimhaiah

**Affiliations:** ^1^Taluka Health Office, Chikkaballapur, Karnataka, India; ^2^ESIC Medical College and PGIMSR, Bengaluru, Karnataka, India; ^3^Center for Operational Research, International Union against Tuberculosis and Lung Disease, New Delhi, India; ^4^District Tuberculosis Office, Chikkaballapur, Karnataka, India; ^5^Department of Community Medicine, Yenepoya Medical College, Yenepoya (Deemed to be University), Mangaluru, Karnataka, India; ^6^National Tuberculosis Institute, Bengaluru, Karnataka, India

## Abstract

**Background:**

In India, as per the latest diagnostic algorithm, all persons with presumptive pulmonary TB (PPTB) are required to undergo sputum smear examination and chest radiography (CXR) upfront. Those with sputum smear positive, sputum smear negative, but CXR lesions suggestive of TB or those with strong clinical suspicion of TB are expected to undergo Xpert MTB/RIF® assay test (also known as CB-NAAT (cartridge-based nucleic acid amplification test)).

**Objective:**

To assess what proportion of PPTB who are undergoing sputum smear examination at microscopy centers of public health facilities have undergone CXR and CB-NAAT. To explore the barriers for uptake of CXR and CB-NAAT from the public health care provider's perspective.

**Methods:**

We conducted a sequential explanatory mixed-methods study in Chikkaballapur district of Karnataka State, South India. The quantitative component involved a review of records of PPTB who had undergone sputum smear examination in a representative sample of seven microscopy centers. The qualitative component involved key informant interviews with four medical officers and group interviews with 9 paramedical staff.

**Results:**

In February and March 2019, about 732 PPTB had undergone smear examination. Of these, 301 (41%) had undergone CXR and 49 (7%) had undergone CB-NAAT. The proportion of PPTB who had undergone CXR varied across the seven microscopy centers (0% to 89%). CB-NAAT was higher in PPTB from urban areas when compared to rural areas (8% vs. 3%) and in those who were smear positive when compared to smear negative (65% vs. 2%). The major barriers for CXR and CB-NAAT were nonavailability of these tests at all microscopy centers and patients' reluctance to travel to the facilities where CXR and CB-NAAT services are available.

**Conclusions:**

CXR and CB-NAAT of PPTB are suboptimal. RNTCP should undertake measures to address these gaps in implementing its latest diagnostic algorithm.

## 1. Introduction

India, with an annual incidence of more than 2.8 million tuberculosis (TB) cases in 2017, is the highest TB burden country in the world. India also has the highest burden of multidrug-resistant TB in the world (~130,000 cases per year) [1]. The government of India's Revised National TB Control Programme (RNTCP) has developed a national strategic plan (2017-2025) for eliminating TB in India by 2025 [2]. For TB elimination, it is essential to diagnose all cases early and provide appropriate treatment. Previous studies have shown that though a large proportion of persons with presumptive pulmonary TB (PPTB) seek health care from public health facilities, many are being missed due to inadequate or incorrect use of TB diagnostic tests [3]. In 2017, RNTCP introduced a revised diagnostic algorithm (also called integrated diagnostic algorithm) for early detection of pulmonary TB and rifampicin-resistant TB [4]. According to this algorithm, all PPTB (with the exception of children and HIV-positive individuals) are expected to initially undergo two basic tests—sputum smear microscopy and chest radiography (CXR). Those with sputum smear positive, sputum smear negative but CXR lesions suggestive of TB or those with strong clinical suspicion of TB are expected to undergo Xpert MTB/RIF® assay test (also known as CB-NAAT (cartridge based nucleic acid amplification test)). CB-NAAT has higher sensitivity and specificity for detecting TB, and it also detects rifampicin resistance [5].

As in several other high TB burden countries, in India, within the public health system, sputum smear microscopy, CXR, and CB-NAAT are not available at all the health facilities [6]. Patients with presumptive TB who seek health care from health facilities without any of these diagnostic tests are referred to undergo these tests at the nearest public health facility (where these tests are available) or patients may choose to undergo these tests at private health facilities. Even in health facilities which have these diagnostic tests, patients may not be prescribed with the tests [7]. The diagnostic tests at the public health facilities are free of cost to the patients, whereas patients may incur out-of-pocket expenditure if they undergo these tests at a private health facility. Therefore, there is a potential for loosing PPTB during diagnosis or PPTB may experience inadequate use of diagnostic tests [8].

Given this situation, it is imperative for the RNTCP to routinely assess what proportion of presumptive pulmonary TB patients seeking care at various health facilities is able to undergo the diagnostic tests as per the latest diagnostic algorithm. There have not yet been any recent research studies on this aspect from any part of the country. In addition, the facilitators and barriers for the service providers to get the diagnostic tests done for their patients may or may not be uniform across various types of health facilities in the Indian public health system. Documenting the context-specific gaps in testing, the possible reasons and solutions to address these gaps from health providers' perspective will help the programme managers and policy makers to undertake appropriate corrective measures to address these gaps. Understanding the magnitude and reasons for gaps in diagnostic testing requires a combination of quantitative and qualitative research methods.

We, therefore, undertook an operational research study in Chikkaballapur district in the South Indian state of Karnataka. The objectives of our study are as follows: (a) to assess the uptake of chest radiography and/or CB-NAAT tests in presumptive pulmonary TB patients who were undergoing sputum smear examination and the demographic and clinical characteristics associated with the uptake of these two diagnostic tests and (b) to explore the facilitators and barriers for the uptake of these tests from the health care providers' perspective.

## 2. Methodology

### 2.1. Study Design

This is a mixed-methods study (sequential explanatory design) where in phase I, the quantitative part (cohort study design) was followed by phase II, the qualitative part (descriptive study design) (reference).

### 2.2. Study Setting

Chikkaballapur district has a population of 1.25 million people (2018) [9]. It is a predominantly rural district located in the south eastern part of the Karnataka State. The public health facilities in the district include primary health centers, community health centers, taluka hospitals (secondary level health facilities), and district hospital (tertiary level health facility). At the public health facilities, there are 13 designated microscopy centers (DMCs)—one at the district hospital, four at the taluka level hospitals, and seven at the primary health centers and community health centers. The facilities for chest radiography are available at the district hospital and the 6 taluka hospitals. CB-NAAT facility is available only at the district hospital. Approximately 1200-1500 persons undergo sputum smear microscopy every month at various DMCs in the district.

#### 2.2.1. RNTCP Integrated Diagnostic Algorithm (2017)

**Figure 1:**
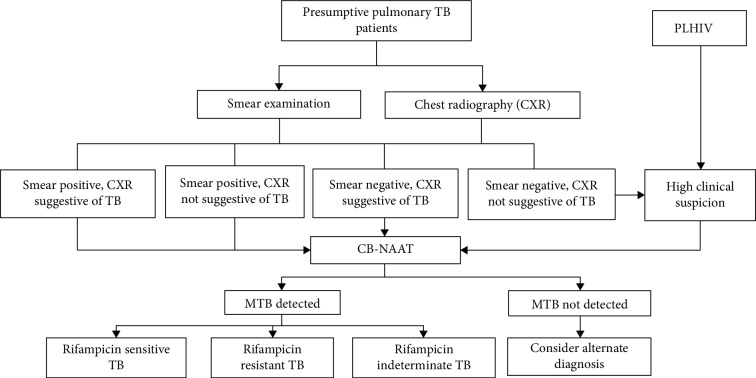
Recommendations for chest radiography and CB-NAAT for the evaluation of presumptive pulmonary TB patients as per the integrated diagnostic algorithm of the Government of India's Revised National TB Control Programme (2017).

The integrated diagnostic algorithm is given in Figure 1. As per this diagnostic algorithm, all presumptive pulmonary TB patients (persons who have cough > 2 weeks or fever > 2 weeks or significant weight loss or hemoptysis) are expected to initially undergo both sputum smear microscopy and CXR. If the sputum smear is positive or if the CXR is suggestive of TB or there is a strong clinical suspicion for TB (even if sputum smear is negative for TB bacilli and CXR is not suggestive of TB), then they are expected to undergo CB-NAAT. Patients attending designated microscopy centers at primary health facilities are referred to undergo chest radiography and CBNAAT. Patients have to physically visit the taluk hospitals or district hospitals for chest radiography. For the CB-NAAT, either the patients have to physically visit the district hospital or in some places the sputum was collected and transported in cold chain by health workers to the district hospital CB-NAAT center. People living with HIV (PLHIV) are eligible to undergo CB-NAAT upfront.

#### 2.2.2. Recording and Reporting System

The demographic and clinical details of all PPTB undergoing sputum smear examination are documented in a laboratory register at each DMC. This laboratory register also contains details of patients referred for CB-NAAT. The details of all patients who undergo CB-NAAT are documented at the CB-NAAT registers maintained at the CB-NAAT site.

### 2.3. Study Population, Study Sites, and Sampling

#### 2.3.1. Quantitative Part

The study population comprised all consecutively enrolled PPTB (aged ≥15 years) who underwent sputum smear microscopy in the months of February and March 2019 at the following seven DMCs: district hospital DMC, two randomly selected Taluka hospital DMCs, and four randomly selected PHC DMCs in the district.

#### 2.3.2. Qualitative Part

The study population for key informant interviews included one physician at a district hospital, one physician at a taluka level, and two doctors at primary health centers, and one group interview included laboratory technicians of seven DMCs and one RNTCP senior TB laboratory supervisor and one senior treatment supervisor.

### 2.4. Study Data Collection Tools, Variables, Sources of Data, and Study Investigators

#### 2.4.1. Quantitative Part

The data on all PPTB who underwent sputum smear examination at the seven selected DMCs were collected on a structured pro forma from the laboratory registers. Data on the following variables are routinely recorded in the laboratory register: date of enrolment, name, age, sex, telephone number, name of the referring health facility, whether the patient belongs to any key populations, results of two sputum smear examinations, HIV status, diabetes mellitus status, and CB-NAAT status. During the study period, we requested the laboratory technicians of the seven DMCs to additionally document whether each patient has already undergone CXR: if not, whether the patient was asked to undergo the CXR and, if yes, from which health facility (public/private) the patient underwent CXR. We cross checked the CB-NAAT notification registers to assess whether the information about the CB-NAAT testing status recorded in the laboratory register was correct or not.

#### 2.4.2. Qualitative Part

We conducted key informant interviews with the medical officers and group interviews with the laboratory technicians STLS and STS, to understand the existing practices with respect to chest radiography and CB-NAAT testing and explore the facilitators and barriers in getting these tests done at the various health facilities. The key informant interviews and group interviews were conducted by two investigators (MK and SBN) who were doctors, have masters in community medicine, and have undergone training in qualitative research methods. An interview guide was developed and used for these interviews. The interviews were conducted in local language (Kannada). The interviews were conducted at a date and place convenient to all the participants and after obtaining their informed consent. We audio recorded all the interviews.

### 2.5. Data Analysis

#### 2.5.1. Quantitative Part

The data collected on a structured format were entered in Epi Data Entry (version 3.1, Epidata Association, Odense, Denmark) and analyzed using Stata Statistical Software (version 15.1, Stata Corporation, College Station, Texas). We described the demographic and clinical characteristics in numbers and proportions. We assessed the number (and proportion) of PPTB who have undergone chest radiography and CB-NAAT. We used the 15^th^ of April 2019 as the cut-off date for assessing the status of whether the PPTB had undergone the chest radiography and CB-NAAT. We used multivariable binomial log models to assess the association between demographic and clinical characteristics with uptake of chest radiography. Since the uptake of CBNAAT was low (<10%), we assessed the association between demographic and clinical characteristics with the uptake of CBNAAT using a chi-squared test or Fischer's exact test (as applicable). A *P* value of <0.05 was considered statistically significant.

#### 2.5.2. Qualitative Part

For interviews with key informants and group interviews, transcripts were prepared the same day based on the verbatim notes/audio recordings. Manual descriptive content analysis was done by the first author (MK). It was reviewed by other co-authors (SS and AKM) to reduce subjectivity in analysis and enhance interpretive credibility. The decision on coding rules and theme generation was done by using standard procedures and in consensus. Any difference between the coauthors was resolved by discussion. The codes/themes were related back to the quantitative data [10], and the findings of the quantitative and qualitative parts of the study have been integrated through narration [11].

## 3. Ethics

We obtained ethics approval from the Ethics Committee of National Tuberculosis Institute, Bengaluru, India (No. A&C/R.2/2019 dated at the 31^st^ of January 2019) and the Ethics Advisory Group of the International Union against Tuberculosis and Lung Disease, Paris, France (No. 121/18 dated at 30.1.19). For the quantitative component of the study, since it is a record-based study, we obtained waiver from obtaining informed consent. For the qualitative part, written informed consent was obtained from all participants.

## 4. Results

### 4.1. Quantitative Part

**Figure 2:**
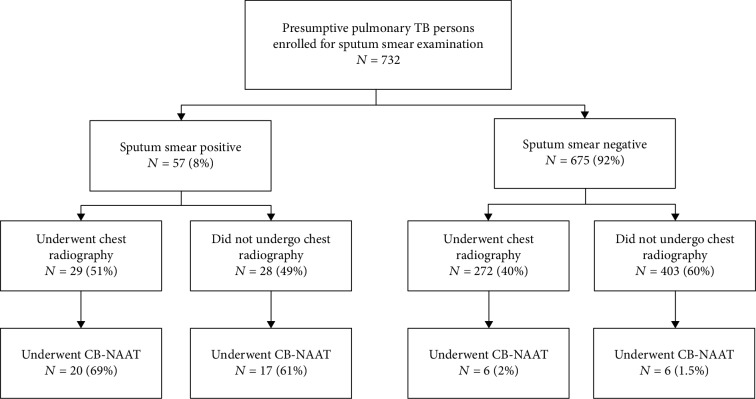
Status of chest radiography (CXR) and CB-NAAT tests in presumptive pulmonary TB patients undergoing sputum smear examination at seven selected designated microscopy centres in Chikkaballapur district, February to March, 2019.

A total number of 732 PPTB were enrolled during the study period; the status of chest radiography and CBNAAT tests among PPTB who underwent smear examination are shown in Figure 2. Their demographic and clinical characteristics are presented in Table 1. About 85% were enrolled from the district and taluka hospitals. The mean age was 46 years (SD 17). Majority (62%) were men, and 73% were living in urban areas. The predominant symptoms were cough and cough with fever, 84% were HIV seronegative, and 70% did not have diabetes mellitus. About 8% were sputum smear positive for acid fast bacillus.

In this cohort, 301 (41%) had undergone CXR with 290 (96%) undergoing it in public health facilities. The association between demographic and clinical characteristics with undergoing CXR is given in Table 2. There were huge variations in the proportion of patients who had undergone CXR across the health facilities (0% in two PHCs to 89% in one of the taluka hospitals). On bivariable analysis, the type of health facility, age of the study participants, residence in urban areas, and having cough, fever, and/or hemoptysis were associated with undergoing CXR. On multivariable analysis, the type of health facility was the only factor that was independently associated with CXR.

Around 49 (7%) of the total 732 PPTB patients had undergone CB-NAAT, and their demographic characteristics are given in Table 3: PPTB who had their residence in urban areas when compared to rural areas (8% vs. 3%) and in those who were smear positive when compared to smear negative (65% vs. 2%).

### 4.2. Qualitative Part

We conducted one group interview with nine RNTCP paramedical workers and four key informant interviews with the medical officers. In these interviews, three facilitators, five barriers, and five solutions were suggested to improve the CXR and CB-NAAT testing.

#### 4.2.1. Facilitators

The following three facilitators were identified:

1. Good awareness about diagnostic algorithm: all lab technicians and senior treatment supervisors and senior Laboratory technician supervisors were aware of the latest diagnostic algorithm
2. Having chest radiography in the same health facility, same day reporting and sharing of results via mobile application were one of the major facilitators for high chest radiography in one of the taluka level hospitals

*“We do X-ray reporting same day and the image is posted in the WhatsApp group of the hospital staff on daily basis…and it helps in same day reporting”* (lab technician)

(3) Doctor insistence for these tests was the third major facilitator for the tests. Patients listen to doctors and get investigation done because of trust and opinion

*“Doctors should tell , patients to get the X-ray done…otherwise they will not go. They have more respect for them [doctors]”* (lab technician)

#### 4.2.2. Barriers

The following five barriers were identified:

1. Nonavailability of the tests at all health facilities: CBNAAT and CXR facility are available only at the district hospital, while only CXR facility is available at taluka hospitals. So patients have to travel some distance to get these basic investigations done

*“In PHC, we don't have x-ray facilities…...even we refer for investigation we cannot trace the patients.”* (senior treatment supervisor)

(2) Patients' reluctance to travel to health facilities or patients have to travel multiple times to get the results: they have to go first to get chest radiography done and then show it to a physician for reporting, and in overcrowded hospitals at taluka and district hospital, it is difficult. Sometimes the concerned doctor (who has to read chest radiology and give reports) may not be available due to other responsibilities of casualty, medicolegal work, and work related to national programmes

*“Patients have to travel twice to get x-ray reports, they will lose work and money for these days, they will simply not go.”* (lab technician at PHC)

*“I do ask x-ray investigation but most of them do not get it done,.. they do not even give their sputum for examination till 3 – 5 repeated visits and symptoms have become more.”* (doctor)

*“In some cases when the sputum is negative and I refer the patient for X-ray/CB-NAAT, they don't listen, they say* “*any ways the sputum test is negative why go so far, we will see later.” This is a big obstacle to convince the patient.”* (doctor)

(3) Long waiting periods: they are long queues at outpatient to consult doctors and followed by waiting at the laboratory for investigations/Chest radiography, reporting and opinion of doctors. Alternate methods of fast tracking PTB patients must be done. Like in some taluka hospitals, they made seals under RNTCP which gives them special access to labs and X-ray labs

*‘If we send the patients to the X-ray unit also…they are not willing to spend time for the investigations due to the long waiting periods'* (lab technician)

*It is very cumbersome for the patient who has to travel from peripheral PHC to taluka and district hospital, even If he/she goes there on time they have to endure the long queues starting from registration, consulting the doctors then up-to the investigations, they get very frustrated and do not come back.”* (doctor)

(4) Charges levied for the chest radiography: at some of the taluka level and district hospitals, they charge INR 100 (~USD 1.5) for any radiographs. This acts as a deterrent for some of the patients to not undergo these tests

*“Patients are charged at the big hospital for X-ray…if the patients directly go to OPD they will be charged Rs 100.”* (lab technician)

(5) Issues with sputum collection and transportation for CB-NAAT tests: the sputum samples produced by patients are of poor quality (i.e., they contain predominantly saliva) or some of them are unable to produce the sputum. Poor quality samples are not accepted for CBNAAT. At times, there were shortages of falcon tubes that can be used for collecting and transporting the sputum specimens

*“How many times do I have to get Kappha (sputum)?” “Patients ask us, some say they do not get sputum even after repeated coughing”* (lab technician)

*“There is shortage of supply of Falcon tubes in the field, so sometimes we are sending in regular sputum cups well packed with ASHA workers, but lab technicians at CBNAAT site are not accepting them”* (doctor)

#### 4.2.3. Suggested Solutions to Improve Chest Radiography and CB-NAAT

We identified the following five suggestions to improve the testing process:

1. Tie-up with private chest radiography centers and CXR vans, to provide CXR facility at PHC level

*“The government should set up under RNTCP mobile X ray units like that of that other health related mobile vans from district hospitals/from NGOs or provide at least small and feasible X ray units at PHC it is a basic investigation requirement and decentralize CB-NAAT by providing it at Taluka hospitals.”* (doctor)

(2) A chest radiography referral slip can be made from PHC so that presumptive pulmonary TB patients have direct/easier access to doctors and laboratory

*“A referral slip with a seal has to be made and should be given to all patients referred from PHCs to x-ray centres for free investigation so they can directly go to the labs….”* (lab technician)

(3) An additional column to be created in the laboratory register for entering details of chest radiography and its results, similar to the columns that are available for documenting CB-NAAT status. This will help in tracking PPTB to undergo these tests
(4) Establishment of a call center at district TB center for tracking and motivating PPTB to undergo investigations and further follow-up
(5) Innovative and appropriate use of technology like using apps on mobile phone and forming groups of lab technicians and physicians for sharing of PPTB information about CXR and CB-NAAT across various health facilities to minimize delays in obtaining patient reports

## 5. Discussion

This is the first study from India to assess the CXR and CB-NAAT of persons with presumptive pulmonary TB undergoing sputum smear examination at various levels of the public health facilities in the country after the introduction of the revised diagnostic algorithm in 2017. The major findings of the study are that only 40% and 7% of the persons with presumptive pulmonary TB have undergone chest radiography and CB-NAAT, respectively, in the study setting.

The strengths of the study are the following: (a) this study was done under routine programmatic conditions in a representative sample of public health facilities in this study district. Therefore, the study findings are reflective of ground level realities in this district; (b) we used a mixed-methods study design to understand the facilitators, barriers, and solutions for improving the uptake of chest radiography and CBNAAT. This has helped us to provide further insights to understand the results of the quantitative part of the study.

The major limitations of the study are as follows: (a) we conducted the study in one district of Karnataka. Therefore, the study findings may not be generalisable to the other districts of the state or in the country. Since Chikkaballapur is one of the better performing districts in the State of Karnataka (one of the states with relatively better health systems in India), we expect the results in other districts to be relatively lower than what we found in our study. (b) Though we requested the laboratory technicians to collect information on the radiological reports of CXR, they could not collect this information systematically in all PPTB who had undergone chest radiography. Therefore, we are unable to comment on how many PPTB had normal radiographs and how many had abnormal radiographs. This has limited our ability to provide information about what proportion of PPTB with radiological abnormalities had had undergone CB-NAAT tests. (c) Due to resource constraints, we did not interview patients to understand their perspectives about undergoing these tests. Therefore, the study is deficient on this aspect. (d) We collected data on the demographic and clinical variables of the study participants that are routinely documented in the RNTCP's sputum smear microscopy laboratory register. We did not collect information on several other variables such as socioeconomic status and educational status that are known to influence whether the person undergoes chest radiography and/or CB-NAAT. We are unable to account for the influence of these variables on our study results.

Despite these limitations, the study findings have the following three major implications on policy and practice:

First, only about 40% of the PPTB had undergone CXR despite the RNTCP diagnostic algorithm recommending upfront CXR for all persons with presumptive pulmonary TB. This is higher than 2% that was reported in another similar study that was done in the year 2012 during the era of previous diagnostic algorithm [12]. In PPTB attending health facilities with CXR services (taluka hospitals and district hospital), the proportion of PPTB who had undergone CXR ranged from 7% to 89%, and in persons attending health facilities without CXR services (primary health centers), the proportion who had undergone CXR ranged from 0% to 42%. This strongly indicates that whether a PPTB undergoes chest radiography or not was strongly dependent on other health facility characteristics and not just on whether the facility offered CXR service or not. Our qualitative interviews indicated that the awareness about the current RNTCP diagnostic algorithm was high among health care providers. The qualitative interviews also indicated that if the medical officer requests the patients to undergo CXR, then patients were more likely to undergo CXR. Therefore, the gaps in the proportion of persons who had undergone CXR are likely to be due to the “know-do” gap; i.e., medical officers were aware of the need for CXR but were not prescribing chest radiography for their patients. This type of “know-do” gap is well documented in previous literature [13, 14]. Apart from this, there were also instances where PPTB charged routine user fees of about INR 100/- to undergo CXR at public health facilities and this may have discouraged them from undergoing this. There are clear guidelines from RNTCP that PPTB be excluded from the payment of the user fees. This aspect needs to be reemphasized to the administrative officials of the hospitals which are charging the user fees so that they do not do so in the future and medical officers prescribing the CXR for persons with PPTB must clearly indicate on their prescriptions that these persons must be exempted from the user fees.

Second, only 7% of this cohort of PPTB had undergone CB-NAAT. Even among those who were sputum smear positive, only 65% had undergone CB-NAAT testing. This indicates severe deficiencies in the provision of CB-NAAT services in the district [15, 16]. Our qualitative interviews indicated that knowledge levels about CB-NAAT among health care providers were high. But the major constraint was having only one CB-NAAT machine in the district to cater to the PPTB from all over the district. Either PPTB had to visit this center to undergo the test or their sputum samples had to be collected and transported by the field workers. It appears that there were challenges in operationalising both these activities at the field level. Given these findings, it appears that improving the CB-NAAT services in the district requires interventions at multiple levels. The exact nature of these interventions and understanding whether they will work in this setting is an area for future research.

Third, the qualitative part of the study indicated some good practices and solutions to improve the uptake of CXR. Notable among these were the practice of forming “WhatsApp®” group to share PPTB information and CXR results among the various healthcare providers (medical officer, laboratory technician, and radiography technician) within the health facility. This facilitated high proportion of PPTB to undergo the chest radiography in one of the taluka hospitals. Similar practices can be adopted in other health facilities. Apart from this, there were suggestions of involving private radiography centers for improving uptake of CXR, usage of a “seal” (to indicate that the person is a presumptive pulmonary TB and needs to be fast-tracked without charging any user fees), and suggestions for creating additional columns in the sputum smear microscopy laboratory register to document the CXR status/results of patients. We agree with these suggestions and recommend these measures for improving the provision of CXR at the field level.

Lastly, our study findings may not be unique and may be found in other settings as well. The gaps in implementation shown in our study have the potential to seriously prevent the realisation of the dream of End-TB in India and around the world [2, 17]. The study findings support the “call for action” for public health community involved in TB eradication in India and at the global level to undertake measures to mitigate the gaps in implementing the existing guidelines on TB diagnostic tests in an equitable manner at the field level [18].

In conclusion, this study shows that chest radiography and CB-NAAT are low. The study provides information of facilitators, barriers, and some solutions for addressing the gaps. We call upon RNTCP to take note of these findings and undertake measures to improve the provision of chest radiography and CB-NAAT services for persons with presumptive pulmonary TB in the country.

## Figures and Tables

**Table 1:** Characteristics of persons with presumptive pulmonary TB attending seven selected designated microscopy centers in Chikkaballapur District during February-March 2019.

Characteristics	*N* = 732	(%)
Designated microscopy center		
District hospital	243	(33.2)
Taluka hospital 1	249	(34.0)
Taluka hospital 2	125	(17.1)
Primary health center 1	32	(4.4)
Primary health center 2	33	(4.5)
Primary health center 3	17	(2.3)
Primary health center 4	33	(4.5)

Age (in years)		
<25	82	(11.2)
25 to 34	138	(18.9)
35 to 44	106	(14.5)
45 to 54	108	(14.8)
55 to 64	129	(17.6)
> 64	146	(19.9)
Not recorded	23	(3.1)

Gender		
Male	455	(62.2)
Female	276	(37.7)
Transgender	1	(0.1)
Place of residence		
Urban	535	(73.1)
Rural	197	(26.9)

Predominant symptom		
Cough	272	(37.2)
Cough+fever	259	(35.4)
Hemoptysis	1	(0.1)
Not recorded	200	(27.3)

HIV status		
Nonreactive	613	(83.7)
Reactive	3	(0.4)
Not recorded	116	(15.8)

Diabetes mellitus		
Yes	8	(1.1)
No	506	(69.1)
Not recorded	218	(29.8)

Sputum examination results		
Negative	675	(92.2)
Positive	57	(7.8)

**Table 2:** Factors associated with undergoing chest radiography in presumptive pulmonary TB persons enrolled for sputum smear examination in seven selected designated microscopy centers in Chikkaballapur District during February-March 2019.

Characteristics	N	n	(%)	RR	(95% CI)	Adj RR	(95% CI)	*P* value
Total	732	301	(41.1)					

Designated microscopy center								
District hospital	243	53	(21.8)	Reference		Reference		
Taluka hospital 1	249	222	(89.2)	4.08	(3.20-5.20)	2.29	(1.01-5.22)	0.047
Taluka hospital 2	125	9	(7.2)	0.33	(0.16-0.64)	0.33	(0.10-1.04)	0.059
Primary health center 1	32	3	(9.4)	0.42	(0.14-1.29)	0.39	(0.12-1.28)	0.122
Primary health center 2	33	0	(0.0)	NE				
Primary health center 3	17	0	(0.0)	NE				
Primary health center 4	33	14	(42.4)	1.94	(1.22-3.09)	1.13	(0.46-2.81)	0.776

Age (in years)								
<25	82	39	(47.6)	0.84	(0.64-1.10)	0.94	(0.64-1.39)	0.775
25 to 34	138	78	(56.5)	Reference		Reference		
35 to 44	106	35	(33.0)	0.58	(0.42-0.79)	0.81	(0.53-1.19)	0.281
45 to 54	108	42	(38.9)	0.68	(0.52-0.90)	1.08	(0.74-1.59)	0.664
55 to 64	129	47	(36.4)	0.64	(0.49-0.84)	0.89	(0.61-1.29)	0.558
> 64	146	54	(37.0)	0.65	(0.50-0.84)	0.94	(0.66-1.34)	0.761
Not recorded	23	6	(26.1)	0.46	(0.22-0.93)	0.95	(0.40-2.25)	0.919

Gender								
Male	455	195	(42.9)	Reference		Reference		
Female	276	105	(38.0)	0.88	(0.73-1.06)	0.92	(0.72-1.18)	0.559
Transgender	1	1	(100.0)	NA				

Place of residence								
Urban	535	257	(48.0)	2.15	(1.63-2.83)	NE		
Rural	197	44	(22.3)	Reference		Reference		

Predominant symptom								
Cough	272	55	(20.2)	Reference		Reference		
Cough+fever	259	225	(86.9)	4.29	(3.37-5.46)	1.91	(0.85-4.30)	0.116
Hemoptysis	1	1	(100.0)	4.94	(3.90-6.26)	4.97	(0.67-36.7)	0.115
Not recorded	200	20	(10.0)	0.49	(0.30-0.79)	0.99	(0.39-2.51)	0.985

Sputum examination results								
Negative	675	272	(40.3)	Reference		Reference		
Positive	57	29	(50.9)	1.26	(0.96-1.65)	1.34	(0.91-2.00)	0.135

NA = not applicable (as outcome predicts exposure perfectly); NE = not estimated due to collinearity with designated microscopy center; RR = relative risk; Adj RR = adjusted relative risk; Reference = strata of data considered as reference population for calculation.

**Table 3:** Factors associated with CB-NAAT in presumptive pulmonary TB enrolled for sputum smear examination in seven selected designated microscopy centers in Chikkaballapur District during February-March 2019.

Characteristics	*N*	*n*	(%)	Chi-square test *P* value
Total	732	49	(6.7)	

Designated microscopy center				
District hospital	243	9	(3.7)	0.09
Taluka hospital 1	249	19	(7.6)	
Taluka hospital 2	125	14	(11.2)	
Primary health center 1	32	3	(9.4)	
Primary health center 2	33	0	(0.0)	
Primary health center 3	17	1	(5.9)	
Primary health center 4	33	3	(9.1)	

Age (in years)				
<25	82	5	(6.1)	0.942
25 to 34	138	10	(7.2)	
35 to 44	106	5	(4.7)	
45 to 54	108	9	(8.3)	
55 to 64	129	10	(7.8)	
>64	146	9	(6.2)	
Not recorded	23	1	(4.3)	

Gender				
Male	455	33	(7.3)	0.336
Female	276	15	(5.4)	
Transgender	1	1	(100.0)	

Place of residence				
Urban	535	43	(8.0)	0.017
Rural	197	6	(3.0)	

Predominant symptom				
Cough	272	12	(4.4)	0.09
Cough+fever	259	18	(6.9)	
Not recorded	200	19	(9.5)	

Hemoptysis	1	0	(0.0)	

Sputum smear results				
Negative	675	12	(1.8)	<0.01
Positive	57	37	(64.9)	

Underwent chest radiography				
Yes	301	26	(8.6)	0.079
No	431	23	(5.3)	

^∗^Fischer's exact test; *P* value. ^∗∗^Chi-square test; *P* value.

## Data Availability

The raw data files used to support the findings of this study are available from the corresponding author upon request.
